# Utilization of a Continuous Saliva Suction Oral Appliance in a Bedfast Patient With Muscular Dystrophy: A Case Report

**DOI:** 10.7759/cureus.79896

**Published:** 2025-03-01

**Authors:** Yuki Kojima, Haruhisa Toguchi

**Affiliations:** 1 Anesthesiology, Asahi General Hospital, Asahi, JPN; 2 Dentistry, Toguchi Medical Lab, Asahi, JPN

**Keywords:** aspiration pneumonia, dental devices, home care, muscular dystrophy, oral appliance

## Abstract

A 28-year-old man with muscular dystrophy was bedridden, requiring 24-hour respiratory support with a ventilator. Owing to repeated episodes of aspiration pneumonia, he was fed through a gastrostomy tube. Mouth opening was limited to 1.5 cm, and he received regular house-call dental treatments to maintain oral hygiene. Owing to excessive saliva production, the patient's family performed oral suction every 10-15 min, which placed a significant burden on them. To alleviate this problem, we proposed the use of an oral appliance. We developed a continuous aspiration oral appliance using information obtained from an intraoral scanner and partial impression-taking. After implementing the device, the frequency of suctions decreased significantly, satisfying the patient and his family. Current advances in technology allow for the creation of oral appliances in many cases, including in-home care settings, benefiting older adults and individuals with severe disabilities who require aspiration prevention.

## Introduction

Muscular dystrophy is classified as a genetically inherited degenerative disorder of muscle. Muscular dystrophy has clinical features of progressive muscle weakness and has a dystrophic pathological appearance on muscle biopsy [1]. Aspiration pneumonia is one of the leading causes of death in patients with muscular dystrophy [2]. One of the most severe complications in patients with advanced Duchenne muscular dystrophy (DMD) and motor neuron disease is the progressive weakening of respiratory muscles, leading to respiratory failure. Almost all DMD patients eventually progress to ventilator dependence [3]. Oropharyngeal weakness eventually jeopardizes safe and adequate food intake, leading to the use of a gastric fistula tube owing to the risk of aspiration. These patients continue to be at high risk for pneumonia because of aspiration of oral secretions and the inability to generate optimal cough reflexes. Here, we present a case report on the use of a continuous saliva suction oral appliance (CSSOA) to prevent aspiration in a 28-year-old man with advanced muscular dystrophy requiring 24-hour respiratory support with a ventilator.

## Case presentation

Clinical application of a saliva suction system

In cases of muscular dystrophy, as a countermeasure for saliva aspiration, low-pressure continuous salivary suction using a suction tube is used. However, this method has some shortcomings, including accidental swallowing or ingestion of the suction tube by biting and difficulty holding the suction tube within the oral cavity. Therefore, this method may be difficult to use in patients who move excessively or overnight while the patients are asleep. Namiki et al. reported a device integrating a suction tube and a mouthpiece that was developed to solve these problems in patients with dysphagia [4]. This device was named the "CSSOA" and was developed to prevent saliva aspiration in older patients. The suction tube is detachable from the mouthpiece. The fabrication process of the CSSOA consists of the following three steps (Video 1):

**Video 1:** Continuous saliva suction oral appliance We developed a continuous aspiration oral appliance using information obtained from an intraoral scanner and partial impression-taking. After implementing the device, the frequency of suctions decreased significantly, satisfying both the patient and his family.

Step 1: Fixation of the Metal Rod to the Working Model

An impression of the patient’s dentition is taken using dental alginate impression material, and a working model is created. To provide a groove for the suction tube in the mouthpiece, a 4-mm metal rod is fixed to the working model using dental wax.

Step 2: Pressing the Mouthpiece Sheet (Erkodur, Erkodent, Germany) Onto the Working Model

The mouthpiece sheet is pressed onto the working model using a pressure-forming device (Erkopress, Erkodent, Germany).

Step 3: Integration of the Mouthpiece and Suction Tube

The suction tube is attached to the mouthpiece to complete the CSSOA.

Case illustration

**Figure 1:**
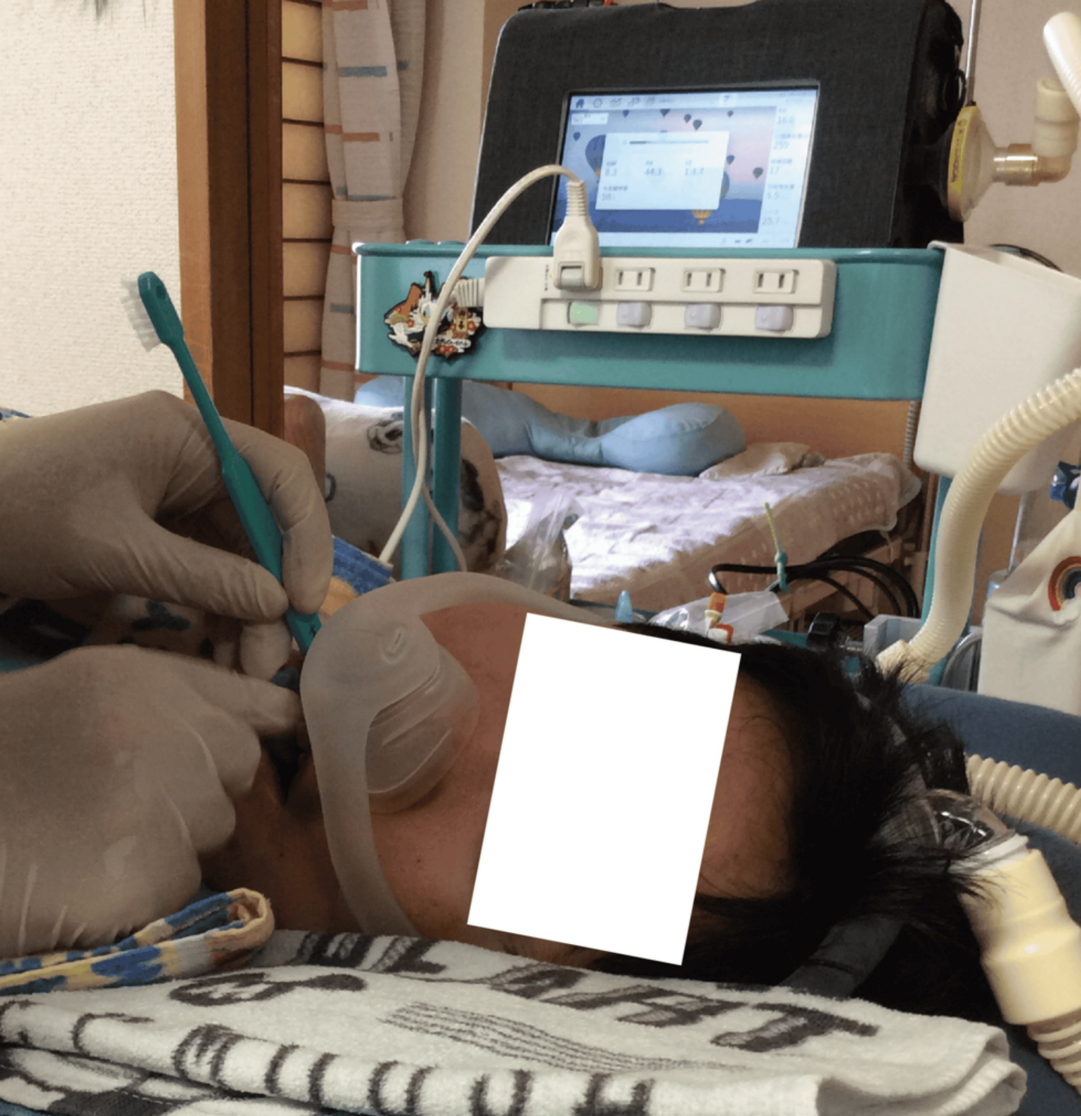
The patient requiring 24-hour respiratory support with a ventilator The patient maintained a lateral position in daily life. Positional changes were performed by family members every day.

**Figure 2:**
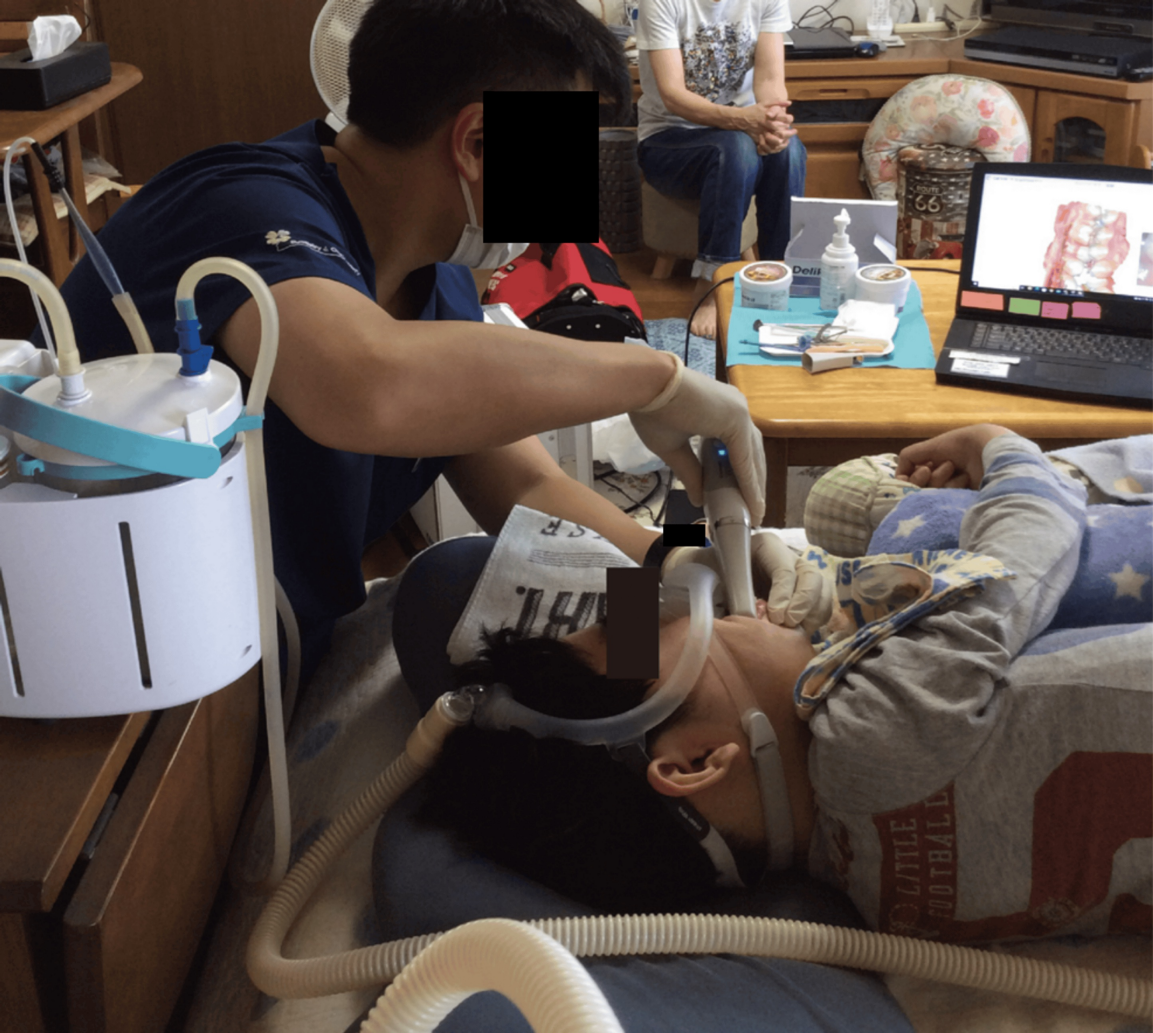
Information obtained from an intraoral scanner and partial impression-taking It was impossible to take an impression of the dentition because of the patient’s narrow mouth opening. Therefore, we made a continuous saliva suction oral appliance (CSSOA) for the patient using an intraoral scanner and partial impression-taking.

A 28-year-old man with muscular dystrophy was bedridden, requiring 24-hour respiratory support with a ventilator (Figure 1). Owing to repeated episodes of aspiration pneumonia, he was fed through a gastrostomy tube. His mouth opening was limited to 1.5 cm, and he had excessive calculus deposition on his teeth. He received regular home-visit dental treatments to maintain oral hygiene. Owing to excessive saliva production, the patient's family performed oral suction every 10-15 min, which placed a significant burden on them. To alleviate this problem, we proposed the use of an oral appliance. However, taking an impression of the dentition was not possible because of the patient’s narrow mouth opening. Therefore, we made a CSSOA for the patient using information obtained from an intraoral scanner and partial impression-taking (Figure 2). After the oral appliance was set, the frequency of suctions decreased significantly in day/nighttime. Although he was still suctioned for phlegm two or three times a day, saliva suction was no longer required while using the oral appliance. The patient, his family, and care staff were satisfied with the device (Video 1).

## Discussion

In many cases, family members serve as the primary caregivers in home medical care for individuals with disabilities, which imposes a significant burden on them [5,6]. Short-term respite care is sometimes available at specialized facilities staffed with healthcare professionals; however, home-based care remains predominant. The burden is not only limited to the medical and nursing staff or the patient’s family but also affects the patient [6]. In the present case, frequent suctioning of saliva posed a considerable burden. The usefulness of a continuous suction mouthpiece during esophagogastroduodenoscopy has been reported [7,8]. However, this pertains only to the procedure itself and does not consider prolonged suction. For the device to be used throughout the day, it is crucial not only to ensure comfort but also to prevent damage to the oral mucosa. Therefore, it is considered appropriate to create a custom device designed by a dentist.

A decade ago, patients with limited mouth opening would have faced considerable challenges in undergoing procedures, such as impression taking. However, with various technological advancements, procedures that were previously deemed impossible are now achievable. A key benefit of this approach is its applicability to individuals with limited mouth opening through the use of an intraoral scanner. Although further studies are required to validate the effectiveness of this device, it has the potential to be used in a wide range of bedfast patients suffering from various neurological disorders.

## Conclusions

This report describes a non-invasive and safe medical procedure to prevent saliva aspiration. After the oral appliance was set, the frequency of suctions decreased significantly. Saliva suction was no longer required while using the oral appliance. The patient and caregivers were satisfied with the device.
